# The Effect of *Bacillus pumilus* 3-19 Metalloproteinase Gene Knockout on the Expression of Minor Proteases of the Strain Degradome and Its PGP Properties

**DOI:** 10.3390/microorganisms14091909

**Published:** 2026-08-28

**Authors:** Damir I. Khasanov, Natalia L. Rudakova, Iuliia V. Danilova, Iuliia A. Vasileva, Aigul I. Gilmutdinova, Margarita R. Sharipova

**Affiliations:** Research Laboratory “Agrobioengineering”, Institute of Fundamental Medicine and Biology, Kazan Federal University, 420008 Kazan, Russia; hasda2149@gmail.com (D.I.K.); danilova146@mail.ru (I.V.D.); vasileva891@mail.ru (I.A.V.); aigwinrygilmyizn@gmail.com (A.I.G.); marsharipova@gmail.com (M.R.S.)

**Keywords:** *Bacillus pumilus*, CRISPR/Cas9, proteases, degradome, metalloproteinases, RT-qPCR, PGPR, barley, *Hordeum vulgare* L.

## Abstract

The *Bacillus pumilus* 3-19 strain exhibits increased secretion of various hydrolases, including proteases, and is a promising plant growth-promoting (PGP) agent. A unique secreted minor metalloproteinase, MprBp, which has no homologs among prokaryotic enzymes, has been identified in its genome. The role of the enzyme in *B. pumilus* cells is poorly understood. Using CRISPR/Cas9 genome editing, a deletion mutant of *B. pumilus* 3-19 with an inactivated mprBp gene was obtained. Comparative analysis of the mutant and native strains by quantitative reverse transcription PCR (RT-qPCR) revealed a significant decrease in the expression of other minor proteases. The mutant strain also demonstrated a two-fold increase in biofilm formation, suggesting that MprBp acts as a negative regulator of this process. Additionally, seed treatment with *B. pumilus* Δ*mprBp* led to an increase in the morphometric parameters of barley seedlings (*Hordeum vulgare* L.).

## 1. Introduction

Modern crop production is facing the challenge of reducing the excessive use of synthetic fertilizers and chemical pesticides, which negatively affect agricultural ecosystems, and finding environmentally friendly alternatives [1]. The uncontrolled use of these agrochemicals has a detrimental effect on the balance of soil ecosystems, leading to decreased fertility, reduced crop yields, and increased susceptibility of crops to both abiotic and biotic stressors.

In this regard, the use of plant growth-promoting rhizobacteria (PGPR) is considered one of the innovative trends to protect and increase the yield of food-important crops. Such properties of PGP bacteria include the ability to perform biological nitrogen fixation, increase the availability of phosphorous and potassium, and synthesize a wide diversity of secondary metabolites, enzymes, volatile compounds, siderophores and others that demonstrate growth-stimulating and phytoprotective activities [2]. Among such microorganisms, representatives of the *Bacillus* genus occupy a special place, characterized by high resistance to adverse environmental factors, production of a broad spectrum of biologically active compounds, and having proven themselves as effective biofertilizers for crop production [3,4,5,6,7,8].

A wide range of *Bacillus* species, including *B. pumilus*, have been identified as potential PGP agents in agricultural research [9,10,11,12]. The researchers’ interest in these bacteria stems from their resilience to various environmental stresses, such as drought, UV radiation, oxidative stress, and exposure to heavy metals. This makes them a promising object for further study to understand their survival mechanisms and to search for potential applications [13,14,15].

Despite numerous studies in recent years, there are still a number of unresolved issues related to the incomplete understanding of molecular and genetic mechanisms that underlie the plant growth-stimulating and biocontrol effects of bacilli, including *B. pumilus*. A comprehensive knowledge of their physiology is essential for the effective use of these microorganisms as PGP strains. One of the key characteristics of *B. pumilus* is its degradome, i.e., the entire spectrum of proteolytic enzymes expressed by the cells [16,17]. However, analysis of the modern literature shows that, despite extensive research on the PGP properties of *B. pumilus*, the role of proteases in these processes has not been fully explored.

The *B. pumilus* 3-19 strain used in this study is a promising PGP agent, derived from the wild-type soil strain *B. pumilus* 7P and having acquired resistance to streptomycin [18,19,20]. One of the phenotypic consequences of induced antibiotic resistance in *B. pumilus* 3-19 is increased production of extracellular hydrolases such as ribonucleases, phosphatases and proteases with potential for commercial use [21,22,23]. Secreted proteases can serve both trophic and regulatory functions, thereby contributing to microbial cell survival [24]. However, the direct role of bacterial secreted proteases in plant–microbe interactions remains poorly understood.

Bioinformatic analysis of the *B. pumilus* 3-19 and *B. pumilus* 7P genomes revealed the identity of their degradomes [19]. The extracellular degradome of both strains is represented by 11 secreted proteases, most of which are proteins with well-characterized functions described in the literature. For example, the protein of biofilm matrix TasA, subtilisin NAT, gamma-glutamyl transpeptidase, inhibitor InhA, etc., have been thoroughly characterized for other members of the *Bacillus* genus [25,26,27,28].

A distinctive feature of these strains is the presence of the gene encoding a minor secreted metalloproteinase, *mprBp*. The uniqueness of this enzyme lies in the absence of homologs among prokaryotic organisms [29,30]. Using the I-TASSER tool (https://zhanggroup.org/I-TASSER/, accesed on 18 August 2026) for protein structure prediction, the closest structural homologs of MprBp were identified as eukaryotic proteases from the adamalysin and astacin families, which belong to the metzincin clan. These enzymes are generally involved in protective and regulatory functions in eukaryotic cells, participating in the processes of intercellular adhesion, cell and tissue differentiation in embryogenesis, extracellular and intracellular signaling, etc. [31,32,33,34,35]. Dysregulation of these enzymes may also be a marker of numerous pathologies.

Previous studies have described the regulatory pathways of MprBp using regulatory mutant *B. subtilis* strains [36,37]. However, the direct biological role of this enzyme in *B. pumilus* cells remains to be elucidated.

In this study, we obtained a mutant *B. pumilus* 3-19 strain with *mprBp* gene deletion using CRISPR/Cas9 genome editing. The key objective of the study was to compare the mutant strain *B. pumilus* Δ*mprBp* with *B. pumilus* 3-19 by analyzing their physiological characteristics and PGP properties, in order to elucidate the role of MprBp not only in *B. pumilus* cells but also in the context of plant–microbe interactions. Additionally, reverse transcription quantitative PCR (RT-qPCR) analysis was performed to assess the expression of *mprBp* and other minor protease genes of interest—the glutamyl endopeptidase (*gseBp*), owing to its distinctive substrate specificity, and the serine proteases (*vprBp* and *eprBp*), which are known to fulfill a wide array of functions in other *Bacillus* species—to identify possible regulatory relationships between MprBp and other components of the *B. pumilus* 3-19 degradome [38,39,40].

## 2. Materials and Methods

### 2.1. Strains and Plasmids

The bacterial strains used in this work are presented in Table 1.

To construct the pKDm06.23 plasmid for metalloproteinase gene deletion, the pJOE9282.1 vector was used (Dr. J. Altenbuchner, Institute of Industrial Genetics, University of Stuttgart, Stuttgart, Germany) [41,42].

### 2.2. DNA Manipulations

Extraction of the genomic DNA of *B. pumilus* 3-19 was performed according to the protocol of the GeneJET Genomic DNA Purification Kit (Thermo Scientific, Vilnius, Lithuania) for Gram-positive bacteria.

Isolation of plasmid DNA from *E. coli* DH5α was performed according to the protocol of the GeneJET Plasmid Miniprep Kit (Thermo Scientific, Vilnius, Lithuania).

Extraction of DNA fragments from agarose gels and PCR-product purification were conducted according to the protocol “Extract DNA from gel using centrifuge” of the GeneJET Gel Extraction Kit and GeneJET PCR Purification Kit (Thermo Scientific, Vilnius, Lithuania).

The quality of purified nucleic acids was evaluated spectrophotometrically using a Nano-500 Mini-Spectrophotometer (Allsheng, Hangzhou, China) and electrophoretically in 1–2% agarose gels stained with GelStain Blue dye (TransGen, Beijing, China).

Sequences of oligonucleotides used for DNA manipulations are presented in Table 2.

Polymerase chain reaction (PCR) was conducted using Taq DNA-polymerase (ELK Biotechnology, Wuhan, China) in a Bio-Rad T100 Thermal Cycler (Bio-Rad, Hercules, CA, USA).

Construction of a plasmid for *mprBp* gene inactivation was carried out according to the protocol [34,35], with the insertion of the sgRNA and flanking regions of the target *mprBp* gene.

The resulting construct, named pKDm06.23, was cloned into *E. coli* DH5α, and its integrity was confirmed by sequencing.

### 2.3. Transformation of B. pumilus 3-19 and Targeted Gene Inactivation by CRISPR/Cas9 Genome Editing

Transformation of* B. pumilus* 3-19 was conducted by electroporation of bacterial cells as described previously by Shen et al. and optimized by Danilova et al. with the use of PEB1 electroporation buffer [43,44]. To enhance transformation efficiency, ampicillin was used as the cell wall-weakening agent, which was added to the *B. pumilus* 3-19 cells at a final concentration of 20 μg/mL 1 h before the required OD600 was obtained [45].

Transformed colonies were plated on LA medium with 0.2% xylose and kanamycin (100 μg/mL) to induce CRISPR/Cas9 genome editing due to xylose-inducible promoter P*_xyl_* and incubated for 48 h at +30 °C [33,34]. Individual colonies were then selected for genomic DNA isolation, and deletion of the target gene was confirmed by conducting a PCR using primers for the *mprBp* gene (listed above).

To eliminate the plasmid, confirmed mutant colonies were incubated for 48 h at +42 °C to prevent its further replication due to the temperature-sensitive origin of replication pE194^ts^ [41,42]. Then the culture was plated on LA medium without antibiotics, and the resulting colonies were tested on kanamycin-supplemented medium to verify plasmid loss. The resulting plasmid-free mutant colonies were additionally confirmed by PCR and sequencing of PCR products.

### 2.4. Examination of Strains’ Growth Dynamics

The growth dynamics of both *B. pumilus* strains was studied for 72 h with measurements taken every 2 h to track changes in optical density at a wavelength of λ = 600 nm using an xMark spectrophotometer (Bio-Rad, Hercules, CA, USA). The strains were cultivated at a temperature of +37 °C with a shaking speed of 180 rpm in 1-L Erlenmeyer flasks containing 300 mL of LB medium (0.5% NaCl) supplemented with streptomycin at a concentration of 500 μg/mL. An overnight 16-h culture was used as the inoculum (1% *v*/*v*).

### 2.5. Proteolytic Activity and Productivity Assay

The overall extracellular proteolytic activity was measured spectrophotometrically by the hydrolysis of azocasein (Sigma, St. Louis, MO, USA) at a wavelength of λ = 450 nm [46]. The activity unit was defined as the amount of enzyme that changed the absorbance by 1 optical density unit per min.

The productivity of the culture was defined as the ratio of the value of overall extracellular proteolytic activity to the value of biomass and expressed in conventional units (c.u.).

### 2.6. RNA Manipulations and Analysis of Relative Minor Protease Gene Expression by RT-qPCR

The total RNA from both *B. pumilus* strains was extracted for RT-qPCR analysis using the RNA Solo kit (Evrogen, Moscow, Russia) from samples taken every 6 h during the study of growth dynamics. The cell pellet was preliminarily lysed using 200 μL of Lysis buffer (20 mM Tris-HCl pH 8.0, 2 mM EDTA, 1.2% Triton X-100) supplemented with lysozyme at a concentration of 20 mg/mL. To eliminate any residual genomic DNA, the RNA samples were treated with DNase I (diaGene, Moscow, Russia).

RT-qPCR was performed using a OneTube RT-PCR SYBR kit (Evrogen, Moscow, Russia) on a CFX96 Touch Real-Time PCR Detection System (Bio-Rad, Hercules, CA, USA). The final concentrations of the components in the reaction were: primers—0.2 μM; reverse transcriptase—1x; test RNA sample—2 ng. Each sample was analyzed in accordance with the MIQE guidelines in three biological and three technical replicates [47]. Every experiment included a negative control without template (NTC) to eliminate the risks of sample contamination or primer dimer formation and a negative control without reverse transcriptase (NRT) to verify the absence of residual genomic DNA.

Housekeeping genes, such as DNA gyrase subunit genes (*gyrA*, *gyrB*), RNA polymerase subunit genes (*rpoB*, *rpoD*) and the *mutL* gene of the mismatch repair pathway, were selected as references. Their primer sequences are shown in Table S1.

Validation of the listed reference genes was performed using the RefFinder program and the built-in algorithms geNorm, NormFinder and BestKeeper [48].

The primer sequences for the studied minor protease genes are presented in Table 3.

The RT-qPCR results were analyzed using Bio-Rad CXF Manager 3.1 software. The relative expression of target genes was calculated using the standard formula 2^−^^(∆∆Ct)^.

### 2.7. Biofilm Formation Assay

Biofilm formation was measured using 96-well plates, following the procedure described by O’Toole et al., in LB medium [49]. Measurements were taken at 12-h intervals.

### 2.8. Motility Assay

Bacterial motility was assessed by placing 5 μL of overnight culture with an OD600 adjusted to a certain value in the center of a Petri dish containing LA medium with 0.3% (*w*/*v*) or 0.6% (*w*/*v*) agar to determine swimming mobility and swarming, respectively [50,51]. Petri dishes were incubated at room temperature, and results were recorded after 24 h of incubation.

### 2.9. Phosphate Solubilization Activity Assay

The ability to solubilize insoluble phosphorus compounds was studied in relation to Ca_3_(PO_4_)_2_ in the National Botanical Research Institute’s Phosphate growth medium (NBRIP) [52]. The Petri dishes were incubated at +30 °C for 7 days. To assess the phosphate-solubilizing activity, the solubilization index (SI) was calculated, defined as the ratio of the sum of the halo zone diameter and the colony diameter (D) to the colony diameter (d).

### 2.10. Production of Indole Compounds

Production of indole compounds was analyzed using the Salkowsky method [53]. The strains were incubated at +30 °C for 48 h in Erlenmeyer flasks containing LB medium (1:4 *v*/*v*) supplemented with 0.4 g/L of L-tryptophan and incubated at 180 rpm. Supernatant samples were collected at 6, 9, 12, 24, 30, 36 and 48 h of incubation. Salkowski reagent (0.05M FeCl_3_ in 35% HCLO_4_) was added to the supernatant (1:4 ratio) and incubated in the dark for 30 min. After incubation, the absorbance was measured spectrophotometrically at a wavelength of λ = 530 nm. Indole-3-butyric acid (IBA) (Sigma-Aldrich, St. Louis, MO, USA) was used as the calibration standard. The results are expressed as IBA equivalents (μg/mL).

### 2.11. Examination of the Strains’ Plant Growth-Promoting Activity on Hordeum vulgare L. Seeds

For the treatment of barley seeds (cultivar “Raushan”) with *B. pumilus* cultures, seeds were vernalized at +4 °C for 24 h. Seed sterilization included soaking in 70% ethanol for 1 min with shaking, followed by soaking in 50% bleach solution with 0.001% Triton X100 added for 30 min with shaking [54]. Afterward, the seeds were rinsed with sterile distilled water until the foam was completely removed.

Seed treatment was performed using a bacterial suspension at a concentration of 1 × 10^9^ CFU/mL for 1 h. Untreated control seeds were soaked in non-inoculated LB medium. The treated seeds were germinated in sterile tall jars (up to 10 seeds per jar), each containing a layer of filter paper on a cotton pad moistened with autoclaved tap water. Germination energy was determined after 3 days; the germination capacity and morphometric parameters of the seedlings (shoot length, root length, and root number) were determined after 7 days of germination.

### 2.12. Statistical Processing of Results

All experiments were performed on three biological replicates, with technical replicates for each biological sample. Data were processed using Statgraphics Plus 5.0 and GraphPad Prism 10.5.0. (673) software to calculate the means *±* SD. Differences between two groups were assessed using Student’s *t*-test, while differences between multiple groups were evaluated using one-way analysis of variance (ANOVA) followed by Tukey’s post hoc test. Differences were considered significant at *p* < 0.05.

## 3. Results and Discussion

### 3.1. Inactivation of mprBp Gene by CRISPR/Cas9 Genome Editing

To inactivate the *B. pumilus* 3-19 metalloproteinase gene, a pKDm06.23 vector was constructed based on pJOE9282.1 with integrated sequences of guide RNA and flanking regions of the target gene (L, R-*mprBp*) (Figure S1A) [41,42]. The integrity of the obtained vector was confirmed by sequencing.

The resulting vector pKDm06.23 was transformed into *B. pumilus* 3-19 cells by electroporation (Figure S1B) [43,44]. To improve transformation efficiency, ampicillin at a concentration of 20 μg/mL was used as a cell wall-weakening agent [45]. Given that many *Bacillus* species are notoriously refractory to transformation, the use of cell wall-weakening compounds, such as glycine, threonine, or Tween 80, can considerably improve this procedure [55]. Nevertheless, transformation efficiency remained low, and all obtained colonies were selected for further analysis.

Induction of the CRISPR/Cas9 genome editing system was performed according to the protocol described in the Materials and Methods section. Deletion of the target gene was verified by PCR using *mprBp*-specific primers, followed by sequencing (Figure S1C).

### 3.2. Examination of Strains’ Growth Dynamics and Accumulation of Proteolytic Activity

**Figure 1:**
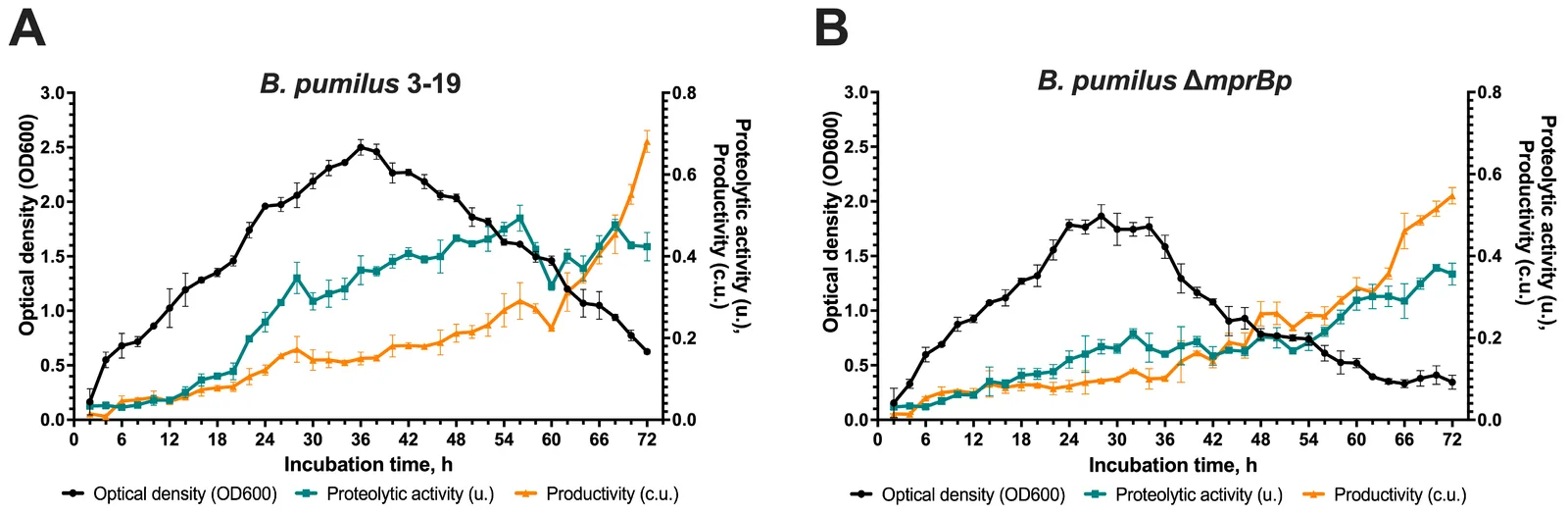
The dynamics of growth and accumulation of proteolytic activity of *B. pumilus* 3-19 (**A**) and *B. pumilus* Δ*mprBp* (**B**).

Examination of the growth dynamics of *B. pumilus* 3-19 revealed that the growth curve did not display the conventional stationary-phase “plateau” (Figure 1A). Maximum cell density was reached at 36 h (OD600 2.45), followed by a gradual decrease. A marked reduction in proteolytic activity was recorded after 60 h of growth, with a subsequent increase that may be explained by the presence of intracellular proteases released into the culture fluid as a result of cell death.

*B. pumilus* Δ*mprBp* showed an approximately 1.5-fold reduction in biomass accumulation compared to *B. pumilus* 3-19, with a sharper decline in optical density beginning at 34 h (Figure 1B). Total extracellular proteolytic activity and productivity decreased nearly 2.5-fold.

These results suggest that MprBp is important for maintaining cell viability in planktonic culture and for extracellular degradome functioning.

### 3.3. Relative Expression of Minor Protease Genes of B. pumilus Strains

The relative expression of minor protease genes was analyzed using RT-qPCR when cultivating strains in a planktonic culture. As reference genes, genes of DNA gyrase subunits *gyrA* and *gyrB*, genes of RNA polymerase subunits *rpoB* and *rpoD* and the mismatch repair gene *mutL* were initially proposed [56,57]. Among the listed reference genes, *gyrB* and *mutL* exhibited the most stable expression and were consequently chosen for normalization in the subsequent analysis of the expression of minor protease genes of interest in *B. pumilus* strains.

**Figure 2:**
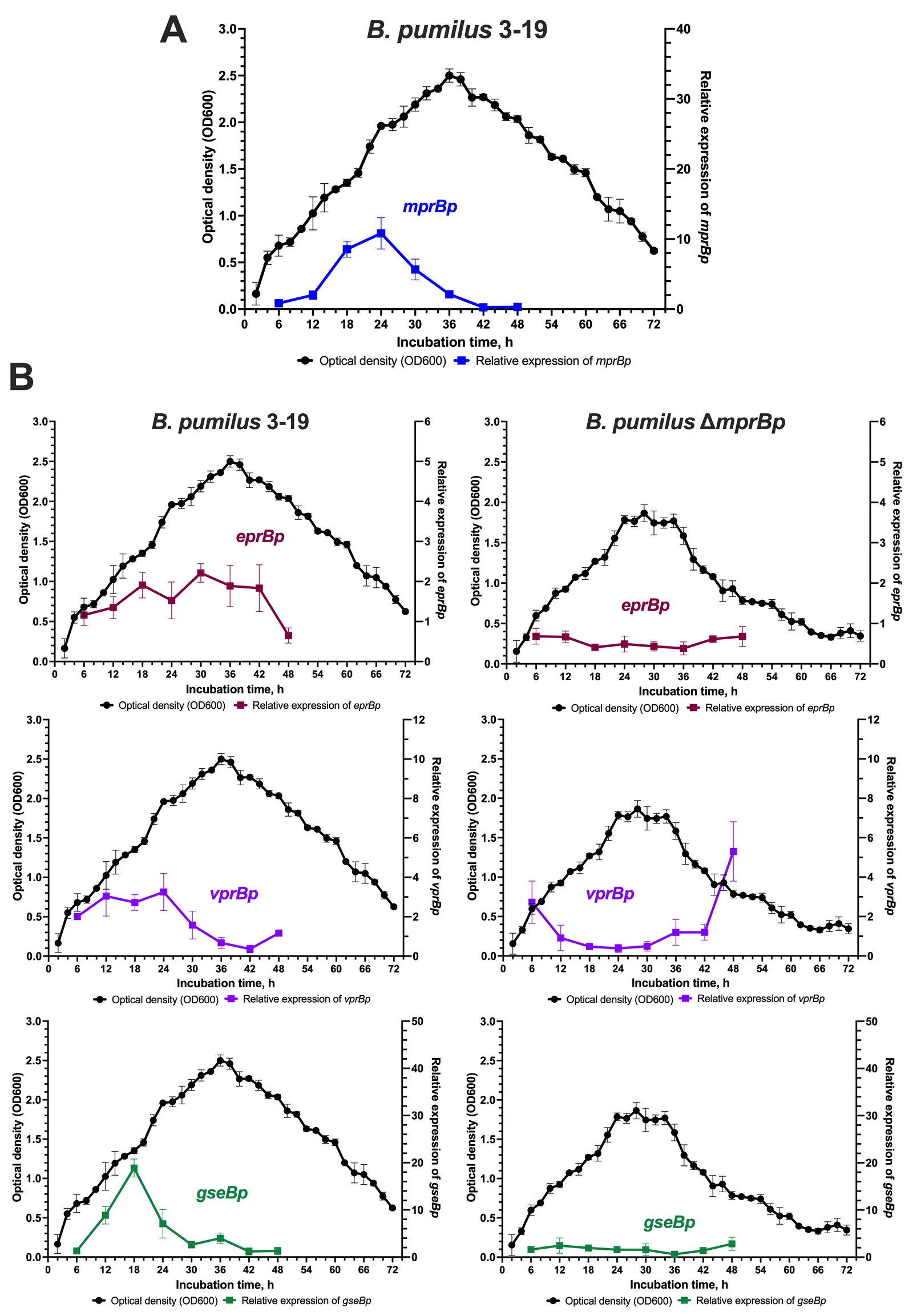
Relative expression of minor proteases of *B. pumilus* strains. (**A**) Relative expression of *mprBp* gene in native *B. pumilus* 3-19 strain. (**B**) Relative expression of minor serine proteases *eprBp*, *vprBp* and glutamyl endopeptidase *gseBp* in *B. pumilus* 3-19 and *B. pumilus* Δ*mprBp*.

We initially examined the expression of the *mprBp* gene in *B. pumilus* 3-19. Expression of *mprBp* was detected between 12 and 42 h, reaching a maximum at 24 h, which may indicate the involvement of this enzyme during the late exponential and early stationary growth phases (Figure 2A). The relative expression levels of the remaining minor protease genes in *B. pumilus* 3-19 and *B. pumilus* Δ*mprBp* are compared in Figure 2B.

The expression of minor serine protease genes *eprBp* and *vprBp* in *B. pumilus* 3-19 was found to be at comparable levels throughout the period of strain active growth. In contrast, expression of *eprBp* in *B. pumilus* Δ*mprBp* was reduced nearly two-fold, while expression of *vprBp* was detected at early hours of growth and at 48 h, suggesting a compensatory role of VprBp.

The expression of *gseBp* in *B. pumilus* 3-19 followed a pattern similar to that of *mprBp* expression, with the peak observed at 18 h. Notably, deletion of *mprBp* resulted in an approximately 10-fold reduction in *gseBp* expression in *B. pumilus* Δ*mprBp.*

These findings point to a complex regulatory network within the extracellular degradome, characterized by mutual interactions between its components, involving potential functional compensation (e.g., expression of *vprBp* in *B. pumilus* Δ*mprBp*) or a decrease in the expression of a potential “partner” (e.g., 10-fold reduction in *gseBp* expression in *B. pumilus* Δ*mprBp*). Further investigation of the expression of other known components of the *B. pumilus* 3-19 extracellular degradome in response to *mprBp* deletion would be of considerable interest.

### 3.4. Biofilm Formation and Motility

Biofilm formation and swarming motility are recognized as multicellular-like behaviors that have been documented in a broad spectrum of microorganisms. These collective behaviors are believed to overlap in its regulatory pathways. In a study by Connelly et al., the impact of deleting extracellular protease genes (including *epr*, *vpr*) on biofilm formation and swarming motility was examined in *B. subtilis* strains [58]. The authors proposed that extracellular proteolytic activity plays a significant role in these types of multicellular-like behaviors. Their results demonstrated that progressive deletion of protease genes led to a gradual impairment in both biofilm formation and swarming motility.

**Figure 3:**
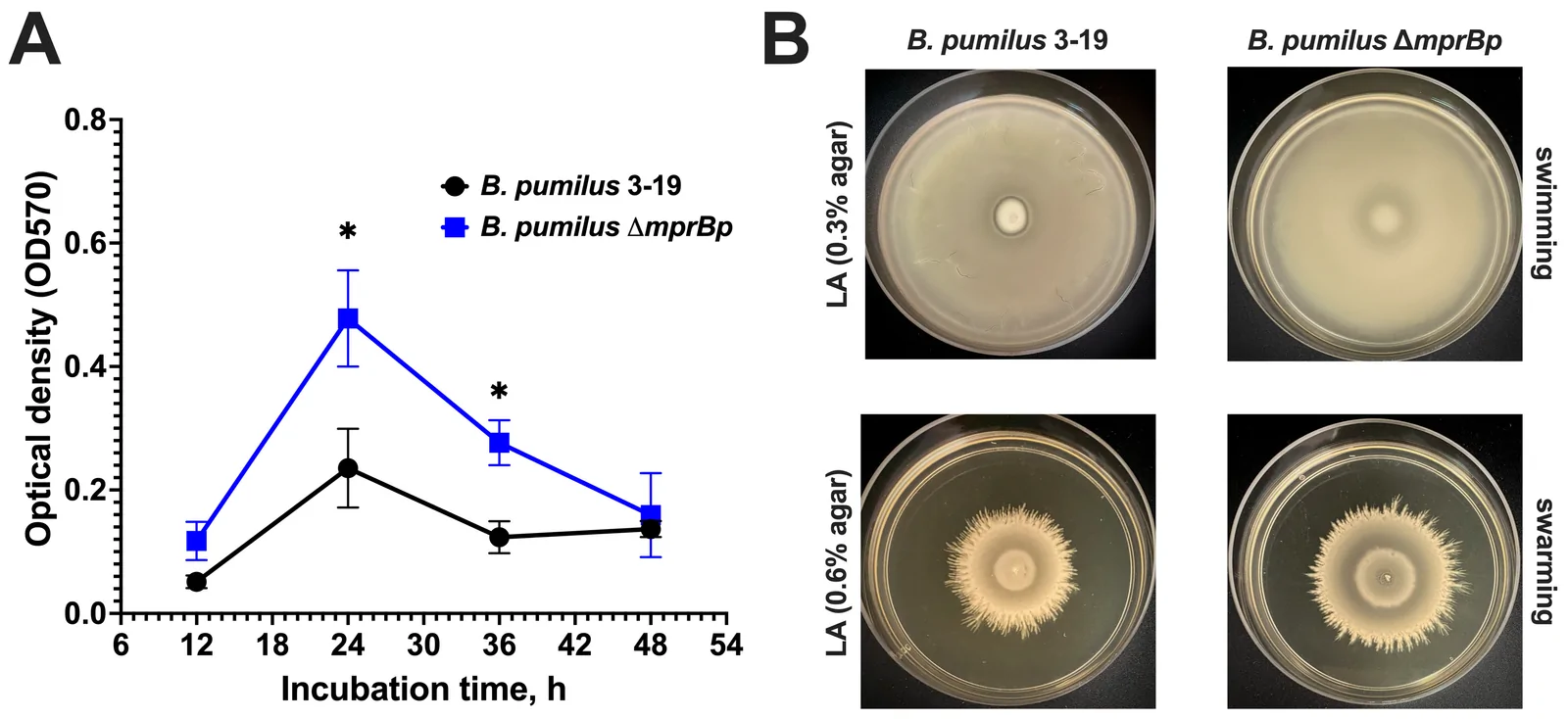
Biofilm formation (**A**) and motility (**B**) of *B. pumilus* strains (* *p* ≤ 0.05).

In the present study, *B. pumilus* Δ*mprBp* exhibited a two-fold increase in biofilm formation compared to *B. pumilus* 3-19 (Figure 3A). By contrast, no significant differences were observed in either swarming motility or swimming (Figure 3B).

Taken together, these findings suggest that MprBp may function as an additional negative regulator of biofilm formation. This regulatory function may also explain the decrease in growth dynamics in planktonic culture of *B. pumilus* Δ*mprBp.*

### 3.5. Examination of the Strains’ Plant Growth-Promoting Activities

Biofilm formation is one of the key traits of PGP strains, enabling microorganisms to colonize plant roots [59]. Given the increase in biofilm formation observed in *B. pumilus* Δ*mprBp*, we decided to investigate whether the deletion of *mprBp* could affect other PGP properties of *B. pumilus* 3-19. To this end, we assessed phosphate solubilization activity and production of IAA. In addition, we evaluated the plant growth-promoting effect of the *B. pumilus* strains on barley seeds (*Hordeum vulgare* L.).

Phosphate solubilization—i.e., the conversion of insoluble phosphates into soluble forms—plays a crucial role in sustainable agriculture by reducing the dependence on phosphate fertilizers. Therefore, the use of phosphate-solubilizing microorganisms (PSMs) is a promising approach in the development of modern biofertilizers [60]. Common PSMs include species from the *Bacillus*, *Pseudomonas*, and *Rhizobium* genera, etc., which are well known for their phosphate-solubilizing activity [60,61].

**Figure 4:**
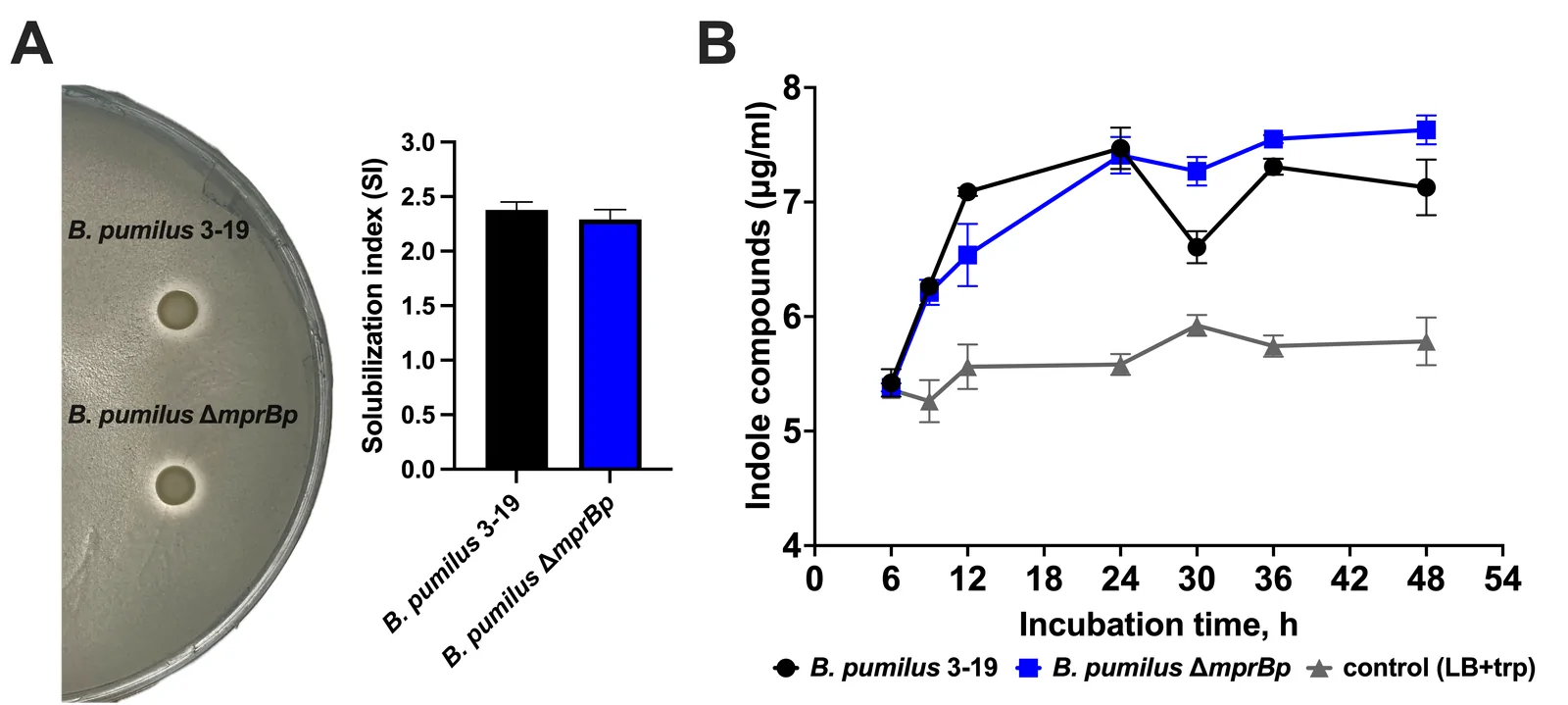
Phosphate solubilization activity (**A**) and production of indole compounds (expressed as IBA equivalents) (**B**) by *B. pumilus* strains.

Phosphate-solubilizing activity was evaluated for *B. pumilus* strains by determining the solubilization index on NBRIP medium supplemented with Ca_3_(PO_4_)_2_. *B. pumilus* Δ*mprBp* demonstrated only an insignificant reduction in phosphate solubilization, indicating that deletion of *mprBp* did not substantially affect this process (Figure 4A).

Production of indole compounds is considered to be a part of the microbial secondary metabolism that depends on the presence of tryptophan in the medium. These auxin-related compounds, including indole-3-acetic acid (IAA), play key roles in plant growth and development [62,63].

To assess the production of these compounds in the studied *B. pumilus* strains, the Salkowsky colorimetric method was used. The mutant strain *B. pumilus* Δ*mprBp* did not exhibit any significant changes in production of indole compounds compared to the native strain. For both *B. pumilus* strains the maximum levels of indole compounds (expressed as IBA equivalents) remained below 7.5 μg/mL (Figure 4B).

The Salkowski method is widely used for screening indole compounds. However, it is known to react with various indole derivatives and may not specifically distinguish IAA from other related compounds [64]. To ensure consistency, we calibrated the assay with IBA and expressed the results as IBA equivalents. The same approach was applied to both strains; therefore, the absence of significant differences between the *B. pumilus* strains indicates that deletion of *mprBp* does not substantially affect the production of indole compounds under the tested conditions.

**Figure 5:**
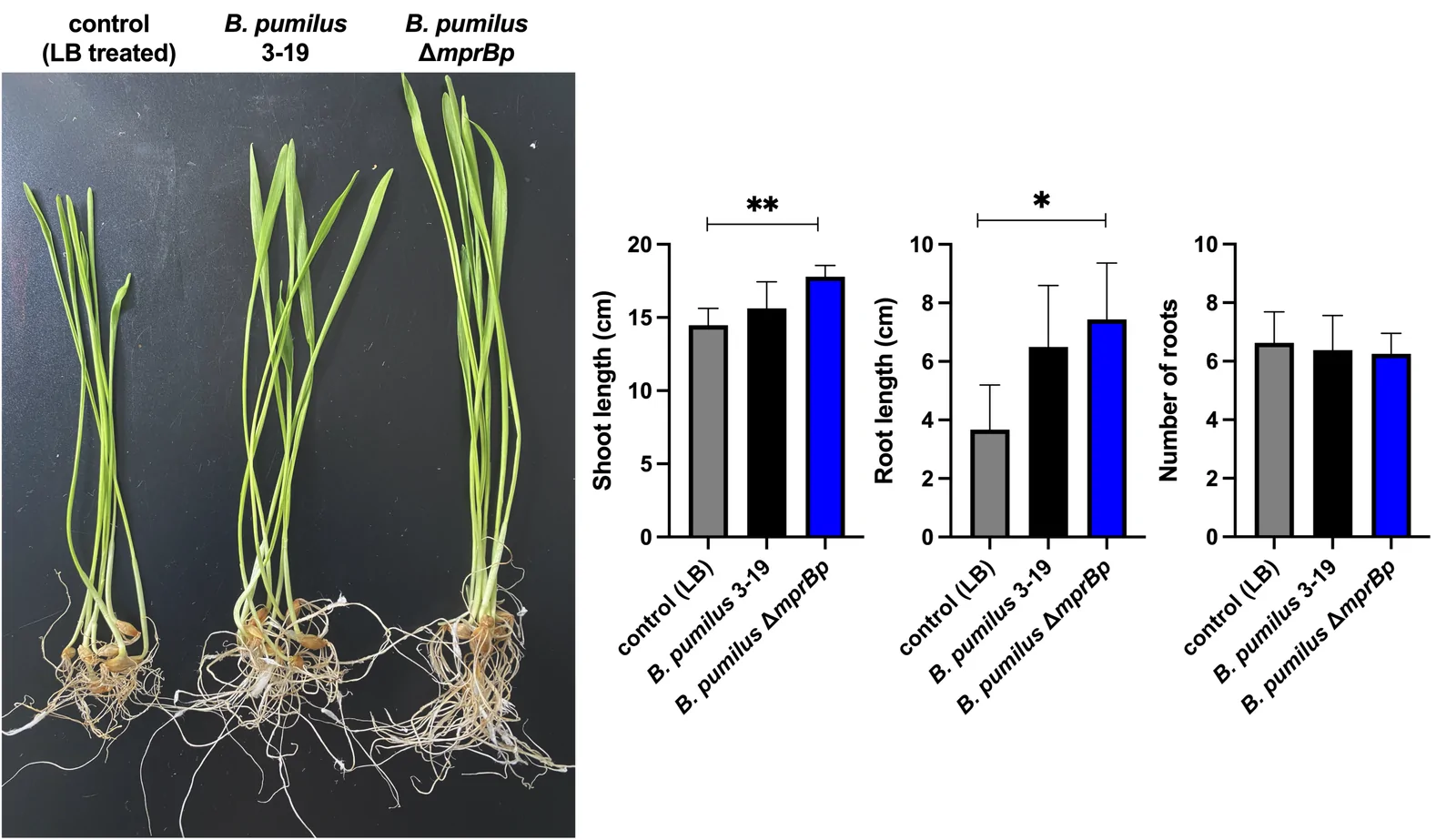
Examination of plant growth-promoting ability of *B. pumilus* strain *in planta* on *Hordeum vulgare* L. (* *p* ≤ 0.05, ** *p* ≤ 0.01).

The growth-promoting activity of *B. pumilus* strains on barley (*Hordeum vulgare* L.) was assessed by determining the morphometric parameters (shoot length, root length, and root number) of seedlings (Figure 5). Treatment with *B. pumilus* 3-19 resulted in a 10% increase in shoot length, whereas treatment with *B. pumilus* Δ*mprBp* led to an increase of nearly 25%. Similarly, the root length increased 1.8-fold and 2-fold, respectively, with an insignificant decrease in the root number.

In summary, the two-fold increase in biofilm formation due to *mprBp* deletion, coupled with unaltered phosphate-solubilizing activity and IAA production, positively affected the shoot and root lengths of *Hordeum vulgare* L. seedlings. These findings create prospects for further in-depth investigation of the PGP potential of *B. pumilus* Δ*mprBp*.

## 4. Conclusions

The mutant *B. pumilus* strain with the inactivated gene encoding the unique minor extracellular metalloproteinase MprBp, constructed using CRISPR/Cas9 genome editing, exhibited statistically significant differences from the parental strain. Comparative analysis of the physiology, the relative expression of minor secreted protease genes, and a limited set of PGP traits in the mutant strain allowed us to establish that the metalloproteinase MprBp plays an important regulatory role in the bacterial cell. In this study, this enzyme can be characterized as a potential negative regulator of biofilm formation. Given that the role of minor proteases can often be highly variable depending on environmental conditions, further studies comparing the parental *B. pumilus* 3-19 strain with the deletion mutant *B. pumilus* Δ*mprBp* obtained in this work are needed, for example, under various abiotic and biotic stresses, as well as in the context of plant–microbe interactions. The results of such studies would help to further characterize the role of the metalloproteinase MprBp in the bacterial cell.

## Figures and Tables

**Table 1:** Bacterial strains used in this work.

Strain	Genotype	Purpose of Use	Source
*Bacillus pumilus* 3-19 [18,19]; Genbank accession number NZ_CP054310	*strR*	Genome editing, recipient strain	Collection of microorganisms from “Agrobioengineering” research laboratory (Institute of Fundamental Medicine and Biology, KFU)
*Escherichia coli* DH5α; https://www.ncbi.nlm.nih.gov/nuccore/NZ_CP076470.1 (accessed on 18 April 2026)	-	Construction of plasmid for *mprBp* gene deletion	
*Bacillus pumilus* Δ*mprBp*	*strR*, Δ*mprBp*	Comparative assay of physiological traits with native *B. pumilus* 3-19 strain	Obtained in this work

**Table 2:** Oligonucleotides used in this work for DNA manipulations.

Primer Name	Sequence 5′→3′ *	Tm (°C)	PCR-Product Length (bp)	Purpose
*sgRNA-mprBp-For*	tacgATCGTGCGAAATATAGTGAC	-	24	For obtaining sgRNA for insertion in pJOE9282.1 preliminarily cleaved by BsaI restriction enzymes
*sgRNA-mprBp-Rev*	aaacGTCACTATATTTCGCACGAT			
*mprBp-L-For*	aaGGCCaacgaGGCCCGTTTCCTAGAGCTCGTCTCC	68	533	To obtain flanking target gene sequences for insertion in the SfiI site in preliminarily cleaved pJOE9282.1 + sgRNA
*mprBp-L-Rev*	aaGGCCatgttGGCCTCTCGTTCAATTGGAGGTGCT			
*mprBp-R-For*	aaGGCCaacatGGCCAGCGGCATTGTCTGCTTAATG	68	497	
*mprBp-R-Rev*	aaGGCCttattGGCCGCAACAACAAGTGTAATGGCGA			
*MprBp-strF*	TGTGAAAATCTAAGGAGGGATAGGAATGAAAAAG CGTTCAG	63	838	To confirm the deletion of the *mprBp* gene
*MprBp-strR*	ATCGAAGCTTTCTGTACCACGCTTTGTTACGG			

Note: * Restriction sites for BsaI and SfiI restriction enzymes are indicated by lowercase letters.

**Table 3:** Primer sequences of the minor protease genes under study and used in this work for RT-qPCR.

Gene Name	Primer Sequence 5′→3′		Tm (°C)	PCR-Product Length (bp)	GC (%)
*mprBp*	For	GCATCATTGAGCAAGCGGAC	65.0	132	55.0
	Rev	CAAAGCTGCGCGAAAGGTTA			50.0
*eprBp*	For	GCATCGATTGGGCCATTAGC	65.0	143	55.0
	Rev	CCCATCATTTCCGCTTGCAG			55.0
*vprBp*	For	CGGCTACTCTGGAAAAGGGG	65.0	143	60.0
	Rev	CTTTAGGCGTCTCTTGCGGA			55.0
*gseBp*	For	CGGAAATTCAGGCTCAGCGA	65.0	92	55.0
	Rev	GACCGCCATTAATCGTTCCG			55.0

## Data Availability

The original contributions presented in this study are included in the article/Supplementary Material. Further inquiries can be directed to the corresponding author.

## Supplementary Materials

The following supporting information can be downloaded at https://www.mdpi.com/article/10.3390/microorganisms14091909/s1. Table S1: Primers sequences of selected reference genes used in the work for RT-qPCR; Figure S1: Inactivation of the *mprBp* gene by CRISPR/Cas9 genome editing. (A): A genetic map of the constructed pKDm06.23 vector, which contains the sgRNA sequence and flanking sequences of the mprBp gene, integrated at the BsaI and SfiI restriction sites (map designed using SnapGene Viewer Version 8.2.1). (B): Electropherogram of PCR-products obtained from *B. pumilus* 3-19 colonies confirming the transformation with the pKDm06.23 vector using primers for the *mprBp* gene: M—Step50 plus DNA ladder (Biolabmix, Novosibirsk, Russia); 1–3—PCR-products of the *mprBp* gene from transformed *B. pumilus* 3-19 colonies (838 bp) and PCR-products amplified from plasmid DNA (328 bp); 4—positive control from the genomic DNA of *B. pumilus* 3-19 (838 bp); 5—negative control. (C): Electropherogram of PCR-products obtained from genomic DNA confirming the deletion of the *mprBp* gene: M—Step50 plus DNA ladder (Biolabmix, Novosibirsk, Russia); 1–3—PCR products amplified from the obtained *B. pumilus* Δ*mprBp* mutant strain (328 bp); 4—positive control from the pKDm06.23 vector used for the transformation of *B. pumilus* 3-19 (328 bp); 5—positive control from the genomic DNA of *B. pumilus* 3-19 (838 bp); 6—negative control.
