# Supplementary material for: The Effect of Bacillus pumilus 3-19 Metalloproteinase Gene Knockout on the Expression of Minor Proteases of the Strain Degradome and Its PGP Properties
Source: Microorganisms. 2026 Aug 28;14(9):1909. doi: 10.3390/microorganisms14091909 (PMC13608939; doi:10.3390/microorganisms14091909)
Supplement: Supplementary file 1 [file microorganisms-14-01909-s001.zip › microorganisms-4495256-supplementary.pdf]

Table S1. Primers sequences of selected reference genes used in the work for RT-qPCR.

Gene name		Primer sequence 5′→3′	Tm (°C)	PCR-product length (bp)	GC (%)
gyrA	For	TTTCCGCAGTTTGAGAGCCT	65.0	193	50.0
	Rev	CAGCCGAAAGTCTTGTCCCT			55.0
gyrB	For	AATCCCGCGGCCATTATCTT	65.0	151	50.0
	Rev	CGGGTCAACCAGTGCAAAAG			55.0
rpoB	For	TCTTTTTCCAGCGTGTTGCG	65.0	122	50.0
	Rev	ATCGGTCGCACACGTAAGTT			50.0
rpoD	For	GCGACATCTCCATCTGACCA	65.0	122	55.0
	Rev	CCGTCATCTAGTCCGAAGCG			60.0
mutL	For	GTTCCAGCTCCTTCACCAGT	65.0	144	55.0
	Rev	GCGCCATGCAACGAGTAAAA			55.0

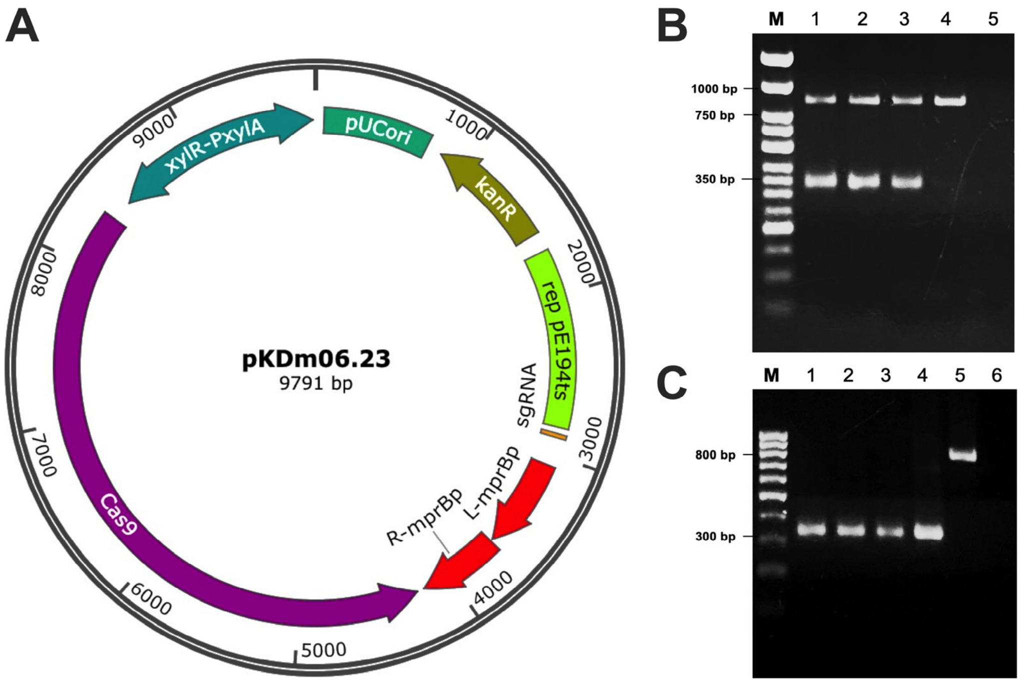

Figure S1. Inactivation of the *mprBp* gene by CRISPR/Cas9 genome editing. (A): A genetic map of the constructed pKDm06.23 vector, which contains the sgRNA sequence and flanking sequences of the mprBp gene, integrated at the BsaI and SfiI restriction sites (map designed using SnapGene Viewer Version 8.2.1). (B): Electropherogram of PCR-products obtained from *B. pumilus* 3-19 colonies confirming the transformation with the pKDm06.23 vector using primers for the *mprBp* gene: M—Step50 plus DNA ladder (Biolabmix, Novosibirsk, Russia); 1-3—PCR-products of the *mprBp* gene from transformed *B. pumilus* 3-19 colonies (838 bp) and PCR-products amplified from plasmid DNA (328 bp); 4—positive control from the genomic DNA of *B. pumilus* 3-19 (838 bp); 5—negative control. (C): Electropherogram of PCR-products obtained from genomic DNA confirming the deletion of the *mprBp* gene: M—Step50 plus DNA ladder (Biolabmix, Russia); 1-3—PCR products amplified from the obtained *B. pumilus* Δ*mprBp* mutant strain (328 bp); 4—positive control from the pKDm06.23 vector used for the transformation of *B. pumilus* 3-19 (328 bp); 5—positive control from the genomic DNA of *B. pumilus* 3-19 (838 bp); 6—negative control.
